# Rare Earth Elements Accumulation in the Hair of Malagasy Children and Adolescents in Relation to Their Age and Nutritional Status

**DOI:** 10.3390/ijerph19010455

**Published:** 2022-01-01

**Authors:** Magdalena Zielińska-Dawidziak, Magdalena Czlapka-Matyasik, Zofia Wojciechowska, Jędrzej Proch, Ryszard Kowalski, Przemysław Niedzielski

**Affiliations:** 1 Department of Food Biochemistry and Analysis, Poznan University of Life Sciences, Wojska Polskiego 28, 60-637 Poznan, Poland; zofia.wojciechowska2509@gmail.com; 2 Department of Human Nutrition and Dietetics, Poznan University of Life Sciences, Wojska Polskiego 28, 60-637 Poznan, Poland; magdalena.matyasik@up.poznan.pl; 3 Department of Analytical Chemistry, Faculty of Chemistry, Adam Mickiewicz University, 89b Umultowska Street, 61-614 Poznan, Poland; jed.proch@gmail.com (J.P.); pnied@amu.edu.pl (P.N.); 4 Department of Meat Technology, Poznan University of Life Sciences, Wojska Polskiego 28, 60-637 Poznan, Poland; ryszard.kowalski@up.poznan.pl

**Keywords:** rare earth elements, nutritional status, Malagasy children and adolescent

## Abstract

Due to undesired influence, the accumulation of rare earth elements (REE) in the human body has been discussed recently. However, it is usually limited to the study of the population living where REE ores and mines are located. The aim of the experiment presented was to analyse the concentration of REE in the hair of children and teenagers living in two areas of Madagascar in relation to the place of residence, nutritional status, age and sex. REE concentration was determined in scalp hair of 262 of subjects (5–19 years old) by an inductively coupled plasma-optical emission spectrometer. The content of total REE in the Malagasy hair was in the range of 0.79–44.15 mg/kg. The nutritional status was evaluated by Cole’s index, and malnutrition of children was observed more often in village areas. The concentration of these elements was also determined in 20 samples for the estimation of environmental exposure. No significant differences were detected in the content of these elements in the studied regions, although the mean value was always higher in soil samples from the Antananariva region. The obtained data suggest dependence between REE concentration in the hair and age, and nutritional status of the examined subjects. Even if the observed correlations are weak, they contribute significant knowledge on the accumulation of REE in the bodies of children living in areas that are not recognised as deposits of these elements.

## 1. Introduction

The application of rare earth elements (REE) in modern industry has increased. They are crucial to the development of the chemical and electronic industry, transportation, health-care, aviation, clean energy, defense and agriculture. The group includes 17 elements with similar features: 15 of lanthanides (lanthanum (La), cerium (Ce), praseodymium (Pr), neodymium (Nd), promethium (Pm), samarium (Sm), europium (Eu), gadolinium(Gd), terbium (Tb), dysprosium (Dy), holmium (Ho), erbium(Er), thulium (Tm), ytterbium (Yb) and lutetium (Lu)) and two from scandium group (scandium (Sc) and yttrium(Y)). Their increased use sparked interest in the impact of these elements on the living organism, particularly on the functioning and development of humans and plants [1].

The largest reserves of these elements are in China, and this country is the leading producer of REE. However, their reserves in African countries, such as Madagascar, are often indicated. Reports on the Ambohimirahavavy ores [2,3] with a concentration of heavy REE (i.e., Eu, Gd, Tb, Dy, Ho, Er, Tm, Yb, Lu and Y), similar to those of the South China ores, also suggest increased exposure of Malagasy human to the effects of these elements.

The biological role of these elements is still poorly recognised. In plants, positive and negative effects have been reported. Some studies have informed about REE accumulation in animal tissue, especially in the liver, bone and lungs [4]. In case of human exposure to REE, cardiac, renal, hepatic and hematological problems are suggested, as well as trouble with bones, gastrointestinal tract, pulmonary, cytogenetic and central nervous system [4,5].

Bearing in mind that children’s nutritional status has an impact on the accumulation of toxic elements in their bodies (research in progress), an attempt was made to investigate the relationship between the place of residence, nutritional status, age and sex; and the level of accumulation of REE in Malagasy bodies. Children living in two areas of Madagascar were analysed: a group from a more industrialised area (the capital, Antananarivo, in the east of country-A) and from a poor town in the agricultural region in the west (near Berevo-B). Compared to the industrialised part of world, the first region is poorly developed, but the important environmental problem is the location of a huge landfill (10 km from the city centre). The second region appears to be completely isolated, and farming practices have remained the same for decades. 

From the standpoint of human health, an important factor is the nutritional status of both studied groups. A human health risk associated with exposure to rare earth elements was studied before by the analysis of REE in the soil and the blood of the human [4,6]. However, hair is considered a very good marker of the accumulation of toxic elements in the human body, especially after long-term exposure [7] and also REE [8,9]. Thus, hair samples were collected to study the level of accumulation of REE in organisms of Malagasy children and adolescent. The hair samples are stable, constant in composition, easy to transport and accumulated elements are not used as a reserve for metabolic processes. They are especially recommended for chronic exposure studies [10,11] and were applied in comparable studies on exposure to REE in China [12,13].

The goals of the presented research were to verify the following research hypotheses: 1. Younger and malnourished children accumulate higher content of REEs in their organisms; 2. neighborhood of unsecured landfill for both municipal and electronic waste increase children’s exposure to toxic REE.

## 2. Materials and Methods

### 2.1. Study Design 

**Figure 1:**
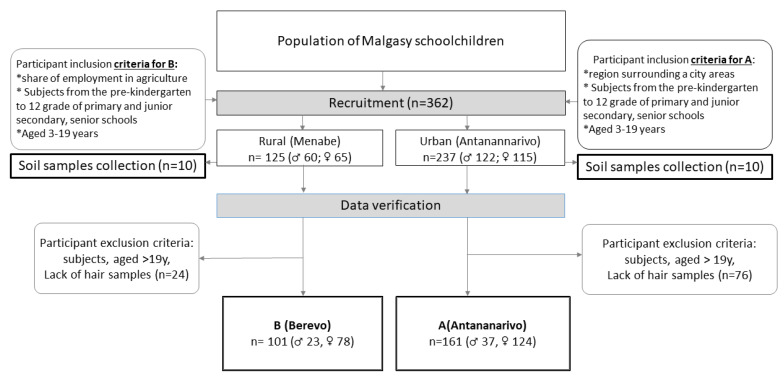
Study design and sample collection (♂ boys and ♀-girls).

The study was conducted in Madagascar in autumn 2018 and involved 262 children and adolescents aged 5–19 years from two regions of the country (study design Figure 1, Table 1). 

The study subjects were recruited (A) close to the capital—Antananarivo—on the east of Madagascar and (B) close to Berevo, on the west of Madagascar, from Menabe regions. Subjects numbering 161 (37 ♂ and 124 ♀) in the A region were enrolled in the study (38 from the center of Antananrivo, 62 from suburban area–Ambohidratrimo—and 61 from the close village Ambatomasina). In the B region, 101 subjects (23 ♂ and 78 ♀) from a school run by a Catholic mission were qualified for the study. At schools in the A region, children and adolescent from the capital and suburban area participated in a feeding program financed by non-governmental organisations. 

The study was conducted according to the guidelines of the Declaration of Helsinki and approved by the Board of Bioethics of Poznan University of Medical Science, Resolution No. 1273/18.

### 2.2. Anthropometrics

Qualified dietitians collected anthropometrical data, body weight (kg) and height (cm) with a precision of 0.1 kg or 0.1 cm, respectively, using professional equipment. According to the guidelines, all measurements were taken in light clothing and without shoes [14]. Obtained results were used to calculate body mass index (BMI, kg/m^2^) was then calculated, and Cole’s Index was nested [15].

(1) $$CI\%=\frac{Weight\;\left[kg\right]\times height\;for\;age\;and\;sex\;{\left(50th\;percentile\right)}^{2}}{Standard\;body\;weight\;\left[kg\right]\times height^{2}}\times 100\%$$

Based on the criteria, the results were interpreted as follows: 140 and above-third-degree obesity, 120–139-second-degree obesity, 110–119-first-degree obesity (overweight), 90–109-recommended values and <90 underweight [14,16,17]. Data are presented in Table 1.

### 2.3. Hair Sample Collection

Hair samples from the occiput area were collected (approximately 1 g). Subjects included in the study had to not have colored or treated hair (dreadlocks, etc.) at least during the last six months. The samples were stored in encoded, sealed polyethylene bags at room temperature until analysis.

### 2.4. Environmental Samples

Samples of soil were collected from regions where children lived (Table 2), from the surface (100 cm^2^). The samples were mixed and stored in Eppendorf-type tubes at room temperature until analysis. 

Two soil types were collected: (1) ferralitic soil (with high level of Al and Fe, and N and P deficiency; dominant in A region), and (2) red ferruginous (dominant in B region).

Samples of water were collected from the same places. They were collected manually and taken from the water supply network (in A region) and from rivers, lakes and wells (in B region). Syringe filters (cut off 0.45 μm) were used for samples’ immediate filtration. They were stored in 40 mL tubes at room temperature. In the laboratory, samples were acidified by nitric acid and stored at ambient temperature until analysis.

### 2.5. Determination of Elemental Composition by Inductively Coupled Plasma-Optical Emission Spectrometers (ICP-OES)

#### 2.5.1. Sample Preparation

Hair samples were washed with acetone and water and dried at ambient temperature. The samples (0.100 ± 0.001 g) were then digested in 5 mL of 65% nitric acid (Merck, Darmstadt, Germany) in closed Teflon containers in the Mars 6 Xpress microwave digestion system (CEM, Matthews, NC, USA) and were filtered through paper filters and diluted with water to a final volume of 15.0 mL. Extraction of the acid leachable fraction from the hair samples was carried out using hydrochloric acid following the previously developed procedure [18]. Extraction of the acid leachable fraction from the environmental samples (1.000 ± 0.001 g of soil or 1.000 ± 0.001 mL of water) was performed with 20 mL 2 M HCL (Merck, Darmstadt, Germany) at 80 °C. Samples were then filtered through paper filters and diluted with water to a final volume of 20.0 mL. Each of the samples was processed in three replicates.

#### 2.5.2. Sample Analysis and Quality Control

An inductively coupled plasma spectrometer with optical emission detection (Agilent 5110 ICP-OES, Agilent, Santa Clara, CA, USA) was used to analyse the samples. The following conditions were maintained for all measurements: plasma gas flow of 12.0 L min^−1^, nebuliser gas flow of 0.7 L min^−1^, auxiliary gas flow of 1.0 L min^−1^ and Radio Frequency (RF) power of 1.2 kW. The most sensitive analytical wavelengths were used (Ce 446.021 nm; Dy 400.045 nm; Er 349.910 nm; Eu 420.504 nm; Gd 342.246 nm; Ho 348.484 nm; La 333.749 nm; Lu 307.760 nm; Nd 406.108 nm; Pr 417.939 nm; Sc 361.383 nm; Sm 442.434 nm; Tb 350.914 nm; Tm 336.261 nm; Y 361.104 nm; Yb 328.937 nm). Potential spectral interferences were recognised in method validation, and background correction methods (fitted or off-peak) were selected. Commercial ICP analytical standards (Romil, England) and demineralised water (Direct-Q system, Millipore, Burlington, MA, USA) were used for calibration. The detection limits were estimated in the range of 0.01–0.09 mg kg^−1^ dry weight (DW) using the criteria of 3-sigma: Ce 0.02; 0.05 Dy; 0.04 Er; 0.07 Eu; 0.07 Gd; 0.06 Ho; 0.02 La; 0.06 Lu; 0.02 Nd; 0.06 Pr; 0.05 Sc; 0.05 Sm; 0.04 Tb; 0.06 Tm; 0.04 Y; and 0.03 Yb mg kg^−1^. The uncertainty level was estimated for the procedure, including sample preparation at the level of 20%. Both certified reference material analyses (soils: CRM and CRM S-1; sediments: CRM 667 and CRM 405; CRM NCSDC (73349)—bush branches and leaves) and standard addition methods were used in quality control with acceptable recovery (80–120%). Aditionally, for selected samples, the analysis using reference analytical techniques (ICP-MS PlasmaQuant MS Q (AnalytikJena, Jena, Germany)) had been performed with acceptable (80–120%) recovery.

### 2.6. Statistical Analysis

On the basis of the studies regarding rare earth elements in human hair (measured in the mining area of China) in subjects between 11 and 77 y and assuming, in turn, 5%, 10% and 15% error of estimation of La and Nd, it was calculated that the minimum sample size in women subgroups equals 61, 50 and 42, and 17, 14 and 12, respectively [12]. A Shapiro–Wilk test evaluated the normality of the distribution of continuous variables in the total sample, A and B regions (skewed variables were logarithmically transformed). For Spearman’s rank correlation and significance (*t*-test) analyses, a significance level of <0.05 was adopted for the threshold. All statistical analyses were performed using STATISTICA software (version 13.0 PL; StatSoft Inc., Tulsa, OK, USA; StatSoft, Cracow, Poland). 

## 3. Results and Discussion

The main source of exposure of the Malagasy population from the studied region to REE seems to be soil, and—as a consequence—consumed food and water harvested from the studied region. The diet of the examined children is based on rice, and for this reason, the deeper layers of soil were not studied—the soil samples were taken from the surface. The examined children were in contact with the soil during everyday functioning.

Table 3 presents the determined content of the REE in the soil samples collected from these two regions (Table 2 shows geographical data).

The average values of REE concentration in the soil samples were significantly lower than those determined for the samples in China for each studied element (except for the Tm) [1,6,13,19] and the Ambohimirahavavy region of Madagascar [2,3]. No significant differences were noted for the content in REE in relation to the location of sample collection (A vs B), although both median and mean value determined were always higher for the samples collected in the more industrialised Antananarivo region. We expected that children from A region could be at greater risk of REE accumulation due to the close location of the unsecured landfill for both municipal and electronic waste. There was a possibility of local transport of pollutants. Hence, the vicinity of the landfill has been distinguished as an area subject to potential anthropogenic pressure. However, our assumption was not confirmed by the studies of environmental samples.

The concentration of REE in collected water samples were below or at the limit of quantification. Therefore, water was not considered as a source of REE for the studied population. The literature proved that REE concentration in soils is higher than in water resources due to cationic exchange capacity and pH action [1,20,21]. REE accumulated in soil bioaccumulate in plants, and this is especially easy in Citrus×limonia, *Oryza sativa* L., *Solanum lycopersicum* L., *Triticum durum* L. and *Vigna radiata* L. i *Zea mays* L. [22,23,24,25,26,27]. Then, REE may enter the food chain.

As REE is supposed to have a significant influence on human health, the accumulation of these elements in the hair of examined children and adolescents were examined in the next stage and presented in Table 4. The least often detected elements (i.e., below the detection level–LOD) in hair were: Tm (not detected—ND in 248 samples), Lu (ND in 223 samples), Ho (ND in 148 samples), Dy (ND in 143 samples) and Er (ND in 122 samples). The highest mean concentrations were observed for light REE, especially Ce, La and Nd (Table 4), as was also noted by Wei et al., 2013. These concentrations were many times lower than those detected in China, both in the mining and longevity region [12,13]. Even the lower levels of REEs may cause human health problems, especially after accumulation in human brain and bones [6]. The total concentration of sixteen studied REEs in Malagasy children hair was in the range of 0.79–44.15 mg/kg (while the mean total concentration was 7.21 ± 7.66 mg/kg).

Previous studies suggest that gender and age influence the concentration of REE in hair [12,13]. At the same time, a higher daily intake with the diet was observed for children, and children are a group more exposed to the neurodegenerative effects of REE on the body (decreasing IQ and memory loss) [28]. Moreover, previous studies found that the nutritional status of girls significantly influenced the accumulation of toxic elements [29]. For this reason, the question of whether there is a relationship between the concentration of REE in the hair of the examined group (children and adolescents) and their gender, age and nutritional status is investigated.

The age range of Malagasy in the study group was 5–19 years (Table 1). Despite the fact that only children and adolescents were examined here, the previously observed tendency [13] was confirmed: The accumulation of most REE (except Ho, Sm and Tb) significantly decreased with the age of individuals (Table 5). Li et al. (2013) explained this by greater consumption of vegetables in relation to other dietary components by children and high bioaccumulation of REE in vegetables [6]. This phenomenon is very disturbing due to the intensive development of the young children, especially their nervous system, and suggested the influence of these elements on it [4,5]. 

The tendency of REE higher accumulation depending sex could not be confirmed due to the insufficient number of samples taken from boys (*n* = 23 in B region), although the trend suggested by Zhang 2020 was observed, i.e., higher accumulation of most of the elements in studied girls’ hair. The average total accumulation of REE in the Malagasy girls’ hair was 7.50 ± 7.99 mg/kg (*n* = 262), while in boys’ hair it was 5.65 ± 7.67 mg/kg (*n* = 60).

Moreover, a significant (although weak or very weak) negative correlation was noted between Cole’s index and accumulation of REE (except Ho, Lu, Sm and Tm) in the hair of children and adolescents (Table 5). This observation confirms a higher susceptibility of malnourished children relative to potential toxic influence of these elements. What is more is that since malnutrition was more often observed in younger children and the accumulation of REE in hair decreased with age, it emphasises the importance of the negative action of REE on developing children. At this stage of the research, authors may speculate only that a diet poor in protein and bioelements and a diet rich in grains increases the accumulation of REE in the bodies of malnourished children. This effect is observed for heavy metals, especially cadmium [30,31,32,33]. Malnourished Malagasy children did not consume meat, fish or milk (nutritional interview data). The only source of protein in their diet and basis of the nutrition in Madagascar is rice, which, as indicated above, bioaccumulate high concentrations of REE from soil [26]. 

Even in the area where the concentration of the studied elements was much lower than in the mining area in China and Madagascar, younger and malnourished children were more susceptible to the activities of REEs. 

The presented results have some limitations. The most important one includes the observed weak correlations, which may arise from a low concentration of REEs in the studied area. However, the research novelty is related to the analysis of the nutritional status of children and adolescents, which has been omitted so far as a factor significant for the accumulation of REE in the bodies of children and teenagers.

## 4. Conclusions

Presented research contradicted the assumption that the concentration of REE in urbanised region A, located close to an unsecured landfill, was higher than in the region completely isolated from the industry, which is the sparsely populated region B of Madagascar. The concentration of REEs in studied soil samples was far below those observed in the mining area. Despite this, it has been found that these elements accumulated in the hair of children; the determined REE concentrations suggest their potential toxic effect. Higher contents of light REE (Ce, La and Nd) were detected in hair samples. Moreover, the results presented confirmed the hypothesis that younger, malnourished children are more susceptible to the accumulation of REE. The effect could be explained by the insufficient supply of essential nutrients in the diet, which causes tissue atrophy. 

## Figures and Tables

**Table 1:** Sample characteristic by sociodemographic and lifestyle variables of A and B groups.

Parameter (Mean ± SD) ^1^	Total Sample (*n* = 262)		East of Madagascar (Close to Antananarivo) (*n* = 161)		West of Madagascar (Menabe Region) (*n* = 101)	
	x ± SD	Min–Max	x ± SD	Min–Max	x ± SD	Min–Max
Age (years)	11 ± 3	5–19	12 ± 4 ^a,1^	5–19	10 ± 3 ^a^	5–16
Weight (kg)	29.2 ± 10.6	13.9–60.7	30.9 ± 11.6 ^a^	13.9–56.1	27.1 ± 8.8 ^b^	14.7–60.7
Height (cm)	134 ± 13	102–167	136 ± 12 ^a^	115–167	133 ± 12 ^a^	102–162
BMI (kg/m^2^)	17.0 ± 2.9	12.1–24.6	17.5 ± 3.1 ^a^	13.3–24.6	16.0 ± 2.1 ^b^	12.1–23.9
Cole index (%)	97 ± 10	79–124	99 ± 10 ^a^	79–124	94 ± 9 ^b^	79–112
Cole index distribution *n* (%) (*p* < 0.001) ^2^:						
Underweight	60 (23)		25 (16)		35 (35)	
Recommended values	174 (66)		113 (70)		61 (60)	
First degree obesity	20 (8)		16 (10)		4 (4)	
Second degree obesity	8 (3)		7 (4)		1 (1)	
The frequency of meals *n* (%):						
Everyday breakfast	161 (62)		161 (100)		0 (0)	
Regular lunch ^3^	90 (34)		90 (56)		0 (0)	
Random meals	133 (51)		32 (20)		101 (100)	
Economic situation of the family ^4^						
Below average	199 (76)		101 (63)		98 (94)	
Average	60 (23)		57 (35)		3 (6)	
Above average:	3 (1)		3 (2)		0 (0)	
Caregiver completed education level:						
Primary or lower	143 (55)		48 (30)		95 (90)	
Secondary	114 (43)		109 (68)		5 (10)	
Upper secondary	5 (2)		5 (3)		1 (0)	
Sources of drinking water:						
Water supply network	161 (62)		161 (100)		0(0)	
Surface water (rivers, lakes, wells)	101 (38)		0 (0)		101 (100)	
Toilets availability at home:						
Yes	161 (62)		161 (100)		0 (0)	
No	101 (38)		0 (0)		101(100)	

^1^ Statistically significant differences between A and B children were marked with different letter inscriptions a,b as *p* < 0.001. ^2^ Statistical significance (Person’s chi-squared test): *p* < 0.0001. ^3^ Participation in the feeding programme at school (1 meal at school). ^4^ The financial situation was assessed using the question: ‘How would you describe your household’s overall situation?’; the ‘modest’ category consisted of two answers: ‘we have to be very careful with our daily budget’ and ‘we have enough money for our daily needs, but we need to budget for bigger purchases’; the ‘comfortably’ category consisted of one answer: ‘we have enough money for our needs without particular budgeting’; the ‘wealthy’ category consisted of one answer: ‘we can afford some luxury’.

**Table 2:** Soil and water sampling sites.

Region	Sample	Geographic Coordinates
A	1	18°49′ S; 47°26′ E
	2	18°48′ S; 47°26′ E
	3	18°47′ S; 47°23′ E
	4	18°45′ S; 47°33′ E
	5	18°49′ S; 47°26′ E
	6	18°56′ S; 48°25′ E
	7	18°55′ S; 47°32′ E
	8	18°55′ S; 47°32′ E
	9	18°55′ S; 47°32′ E
	10	19°39′ S; 46°32′ E
B	11	19°32′ S; 45°27′ E
	12	19°40′ S; 45°23′ E
	13	19°42′ S; 45°20′ E
	14	19°45′ S; 45°2′ E
	15	19°43′ S; 44°58′ E
	16	19°16′ S; 44°57′ E
	17	19°43′ S; 44°57′ E
	18	19°43′ S; 44°57′ E
	19	19°42′ S; 44°34′ E
	20	20°17′ S; 44°16′ E

**Table 3:** Comparison of rare earth elements content in the soil samples (mg/kg).

Element	Place	Content (mg/kg)							*p*
		Median	Min	Max	25th	75th	Mean	SD	
Ce	A	21.82	14.48	50.34	17.62	22.12	25.28	14.37	0.09
	B	13.91	3.62	32.22	8.98	15.42	14.25	8.11	
Dy	A	2.06	1.23	4.41	1.29	2.83	2.37	1.32	0.13
	B	0.99	0.40	3.64	0.69	1.68	1.37	0.98	
Er	A	0.67	0.44	1.24	0.58	0.94	0.77	0.32	0.07
	B	0.41	0.20	0.88	0.27	0.60	0.47	0.25	
Eu	A	0.28	0.10	0.48	0.12	0.33	0.26	0.16	0.21
	B	0.14	0.08	0.36	0.09	0.24	0.17	0.10	
Gd	A	2.50	0.87	4.58	1.27	4.24	2.69	1.68	0.11
	B	0.73	0.07	4.32	0.63	1.89	1.31	1.29	
Ho	A	0.03	<LOD	0.16	<LOD	0.12	0.11	0.07	0.35
	B	0.02	<LOD	0.11	<LOD	0.06	0.04	0.35	
La	A	10.51	6.79	26.48	7.98	11.42	12.64	7.96	0.06
	B	7.16	1.8	11.89	4.61	8.10	6.73	2.89	
Lu	A	<LOD	<LOD	0.19	<LOD	0.16	0.08	0.09	0.44
	B	<LOD	<LOD	0.26	<LOD	<LOD	0.04	0.08	
Nd	A	8.52	4.63	20.47	5.42	10.28	9.86	6.36	0.08
	B	6.33	1.72	8.52	3.92	6.96	5.59	2.11	
Pr	A	4.21	2.13	8.12	2.36	4.38	4.24	2.40	0.08
	B	1.95	0.55	5.47	1.60	2.64	2.31	1.40	
Sc	A	1.73	0.83	3.01	1.00	2.93	1.90	1.03	0.31
	B	0.61	0.37	4.22	0.43	1.53	1.21	1.23	
Sm	A	2.59	1.53	4.31	1.87	2.60	2.58	1.07	0.86
	B	1.64	0.64	2.54	1.47	2.15	1.76	0.59	
Tb	A	0.3	0.01	0.62	0.11	0.60	0.33	0.28	0.17
	B	<LOD	<LOD	0.67	<LOD	0.24	0.14	0.22	
Tm	A	1.77	1.29	2.59	1.76	2.13	1.91	0.49	0.31
	B	1.02	0.38	3.03	0.78	1.89	1.43	0.92	
Y	A	1.09	1.30	6.68	1.85	4.74	3.34	2.29	0.06
	B	2.12	0.71	2.86	1.046	2.15	1.60	0.82	
Yb	A	0.46	0.15	0.73	0.26	0.52	0.42	0.22	0.08
	B	0.15	0.06	0.64	0.07	0.28	0.21	0.19	

LOD value: Ce, Dy—0.023; Er—0.018; Eu, Gd—0.034; Ho, Lu, Tm, Y—0.031; La—0.0086; Nd—0.012; Pr—0.032; Sc—0.024; Sm—0.026; Tb—0.027; Yb—0.014.

**Table 4:** The content of rare earth elements in the hair of studied children and teenagers.

Element	Content (mg/kg)						
	Median	Min	Max	25th	75th	Mean	SD
Ce	1.54	<LOD	101	0.43	4.04	3.02	3.04
Dy	<LOD	<LOD	1.24	<LOD	0.17	0.14	0.22
Er	0.02	<LOD	0.37	<LOD	0.07	0.05	0.06
Eu	0.029	<LOD	0.22	<LOD	0.05	0.04	0.04
Gd	0.26	<LOD	1.42	0.15	0.48	0.35	0.28
Ho	<LOD	<LOD	0.67	<LOD	0.07	0.06	0.09
La	0.75	<LOD	9.29	0.27	2.09	1.38	1.53
Lu	<LOD	<LOD	0.08	<LOD	<LOD	0.39	0.01
Nd	0.64	<LOD	7.44	0.32	1.75	1.17	1.28
Pr	0.35	<LOD	3.19	0.08	0.76	0.52	0.56
Sc	0.13	<LOD	1.36	0.05	0.35	0.23	0.27
Sm	0.20	<LOD	2.13	<LOD	0.49	0.32	0.39
Tb	0.07	<LOD	0.34	<LOD	0.14	0.09	0.0.7
Tm	<LOD	<LOD	0.29	<LOD	<LOD	<LOD	0.03
Y	0.20	<LOD	2.71	0.09	0.53	0.40	0.47
Yb	0.03	<LOD	0.33	<LOD	0.07	0.05	0.05

LOD value: Ce, Dy—0.023; Er—0.018; Eu, Gd—0.034; Ho, Lu, Tm, Y—0.031; La—0.0086; Nd—0,012; Pr—0.032; Sc—0,024; Sm—0.026; Tb—0.027; Yb—0.014.

**Table 5:** The determined correlation coefficients (R) between the concentration of REEs in subjects’ hair (*n* = 262) and their age and Cole’s index.

Element	Correlation Coefficient (R) between the Concentration of REEs in Subjects’ Hair and	
	Age	Cole’ Index
Ce	−0.388	−0.256
Dy	−0.364	−0.216
Er	−0.282	−0.174
Eu	−0.328	−0.216
Gd	−0.384	−0.208
Ho	*0.135*	*0.066*
La	−0.368	−0.256
Lu	−0.178	*0.114*
Nd	−0.357	−0.232
Pr	−0.303	−0.234
Sc	−0.467	−0.283
Sm	*−0.071*	*−0.074*
Tb	*−0.073*	−0.166
Tm	−0.178	*0.086*
Y	−0.346	−0.265
Yb	−0.375	−0.277

Correlation factors which were statistically not significant has been marked by italics.

## Data Availability

The authors of the publication have all the data at their disposal; the data can be shared by the corresponding author.
